# Trapped in a double cage: How patients’ partners experience the diagnosis of advanced cancer in times of the COVID-19 pandemic: An interpretative phenomenological analysis

**DOI:** 10.1177/02692163221080660

**Published:** 2022-03-10

**Authors:** Sophie Opsomer, Sofie Joossens, Jan De Lepeleire, Peter Pype, Emelien Lauwerier

**Affiliations:** 1 Academic Center for General Practice, KU Leuven, Belgium; 2 Department of Public Health and Primary Care, Ghent University, Belgium; 3 Program of Health, University Colleges Leuven—Limburg, Belgium; 4 End-of-Life Care Research Group, Vrije Universiteit Brussel (VUB) and Ghent University, Brussels, Belgium; 5 Department of Experimental Clinical and Health Psychology, Ghent University, Belgium

**Keywords:** COVID-19, pandemics, resilience psychological, caregivers, advanced cancer, palliative care, adaptation psychological, qualitative research

## Abstract

**Background:**

When confronting a partner’s diagnosis of advanced cancer, family caregivers are often protected against severe psychological illness by their mental resilience. However, the current COVID-19 pandemic endangers this resilience through the daily threat of contagion exposure, viral transmission, isolation, and fear of death.

**Aim:**

To examine the experiences of partners caring for a person with advanced cancer during the COVID-19 pandemic.

**Setting:**

Twelve partners (all under the age of 65) of persons newly diagnosed with advanced cancer immediately before or during the pandemic were interviewed. An interpretative phenomenological approach was used in analyzing the data.

**Findings:**

Partners experience the COVID-19 pandemic as “living in a double cage.” Due to pandemic mandates and restrictions, the pace of their lives slows. However, COVID-19 does not slow the progression of the cancer, nor does it allow for an escape from the cancer. The pandemic has a significant impact on several elements of resilience. Nevertheless, the participants succeed in adapting and coping in a balanced and creative way despite the new challenges imposed by the pandemic.

**Conclusion:**

The COVID-19 pandemic challenges one’s resilience, a process that, under normal circumstances, may evolve while caring for a partner diagnosed with advanced cancer. Although most partners seem to cope adaptively with both advanced cancer and COVID-19, healthcare professionals should be aware of the risk of exhaustion. Furthermore, it can be presupposed that threatened, contextual factors that may support resilience should be preserved to increase the chances for a resilient outcome.

**What is already known about the topic?**

- Most informal caregivers adapt well to a family member’s diagnosis of advanced cancer and follow a resilience trajectory throughout caregiving.
- The COVID-19 pandemic can be considered a potentially traumatic event. The pandemic is a community threat that enhances the risk for mental and traumatic stress reactions in individuals.
- It is not known what it means for partners to deal with advanced cancer living under the COVID-19 pandemic.

**What this paper adds**

- Cancer caregiving during the COVID-19 pandemic places an extra burden and may fuel further disconnection with the “outer world.”
- Some resilience predictors are strained, others are stimulated.
- The participants succeed in adapting and coping in a balanced and creative way under the new challenges imposed by the pandemic.

**Implications for practice, theory, and policy**

- Healthcare professionals should be aware of the caregivers being at risk of exhaustion.
- Healthcare professionals should recognize the impact of a second, potentially traumatic event on existing resilience predictors.
- More research is needed to explore the effects on resilience of two or more successive potentially traumatic events.

## Introduction

Advanced cancer, defined as cancer unlikely to be cured, affects millions of people worldwide annually.^1^ The majority of persons with advanced cancer prefer to be cared for at home by a family caregiver, often the partner with whom the patient shares an intimate relationship.^2^ This, however, puts the partners at risk for psychological distress, diminished physical health, and lower quality of life.^3–7^ Nevertheless, while a variety of coping mechanisms might be observed^8^ most are expected to adapt well.^9–12^ This process of adapting when confronted with a potentially traumatic event, such as being the partner of a person diagnosed with advanced cancer, is called resilience.^13,14^

At this time, circumstances are particularly complicated due to COVID-19. All aspects of life are affected both directly by the threat of contracting the virus and indirectly by the measures taken (e.g. lockdown, curfew, and social distancing).^15,16^ As a result, the risk for mental distress, severe psychosocial illness, and traumatic stress reactions has increased.^15^ Nevertheless, most individuals are expected to adapt resiliently.^17–19^ However, for those diagnosed with advanced cancer immediately prior to or during the pandemic, the situation is complicated dramatically. Without warning, patients and partners are expected to deal with a second potentially traumatic event, over and above the potentially traumatic event of the diagnosis of advanced cancer. They face a new reality, namely one characterized by a double threat of exposure to the contagion, viral transmission, isolation, and fear of impending death. It is possible the process of resilience—as observed in most partners—might become hindered or strained. As a result, we would expect most carers to recover more slowly or adapt less successfully to this adversity. This may lead to an increased risk of distress and (mental) health issues. This, though, is a novel situation, and while the pandemic will eventually end, its aftermath will likely be felt for years. Through its threatening nature, it nonetheless offers the unique opportunity to refine the concept of resilience and to discover the challenges to resilience among partners of persons recently diagnosed with advanced cancer. To the best of our knowledge, our team is the first to study the resilience process when challenged by two concurrent potentially traumatic events. During our research, we undertook an exploratory stance and posed the research question as follows: “What are the experiences of partners taking care of a person with advanced cancer during the COVID-19 pandemic?”.

## Methodology

### Study design

The interview data stem from a broad longitudinal study design on the development of resilience in cancer caregiving. As soon as the COVID-19 pandemic hit Belgium, it became clear that dealing with a second potentially traumatic event would greatly influence the development of resilience. The interview data naturally revealed partners’ challenges of coping with a patient’s disease within the context of the COVID-19 pandemic. This highlighted the need for an in-depth analysis of the lived experiences of dealing with two potentially traumatic events simultaneously. Therefore, a qualitative interview study with interpretative phenomenological analysis (IPA) of the data was established, Interpretative phenomenological analysis is a method developed for the in-depth analysis of how people make sense of what is happening, which seemed best suited to analyze our participants’ lived experiences.^20,21^ The participants, who were selected from the original study, form a homogeneous group as is preferable for an interpretative phenomenological analysis. Indeed, they are all adults under 65 years old and are all dealing with two potentially traumatic events at a time: partners having recently been diagnosed with advanced cancer and living under the threat of COVID-19 and its resultant measures.

### Participant selection

Population: As recommended by the interpretative phenomenological analysis (IPA), only a small number of participants meeting the following inclusion criteria were included^20^:

– Being the partner and principal caregiver of a person recently (less than 6 months) diagnosed with cancer in an advanced or palliative stage. Advanced stage cancer is defined as cancer in stage III, IV, or metastatic cancer. Cancer in a palliative stage means that the goal of a cure is no longer reasonable or life expectancy is 1 year or less.
– Adults under 65 years of age.
– Fluency in Dutch.

The exclusion criteria were:

– Partners with diagnosed depression or psychological illness before the cancer diagnosis.
– Partners of patients with a life expectancy of 3 months or less.

Sample: For the purpose of the present study, seventeen semi-structured interviews of the original study on resilience in cancer caregiving were enriched with questions that probe for the experiences under the COVID-19 pandemic. From the interview data, we selected nine interviews that were the richest in terms of our research aim and in line with interpretative phenomenological analysis methodology prescriptions. However, the ninth interview seemed to reveal a new code. Consequently, three more interviews were selected. In spite of this, no new codes could be discovered; it could therefore be assumed that with this sample, data saturation had been reached (see Supplemental material 1).

Recruitment: Carers fitting within the inclusion criteria were given an informative flyer about the longitudinal study by the oncological teams of the university hospitals of Leuven and Ghent and by the general practitioners of the Leuven north regions. This flyer could also be found on the websites of peer groups of those with advanced cancer. Candidate participants contacted the researcher (SO) themselves by e-mail or telephone. Consequently, they received further oral and printed information about the study. After giving written informed consent, the interviews were scheduled.

### Data collection

The study was initiated by the first author (SO)—a family physician experienced in palliative care and qualitative research—as part of her PhD project. The interviews were conducted by the first author (SO) between March 2020 and February 2021. She had neither professional nor personal relationships with the candidates. The interview guide was initially designed to study resilience trajectories in cancer caregiving. For the purpose of the present study, the initial interview guide was enriched by questions related to experiences under the COVID-19 pandemic and its measures. Those interview fragments (along with the fragments in which the participants spontaneously spoke about their experience of providing care in times of COVID-19), formed the dataset for the present study (see Supplemental material 2). Because of the pandemic, all but one interview took place via Zoom. The interviews were video recorded and transcribed verbatim, including all non-verbal gestures.

### Data analysis

**Figure 1.**
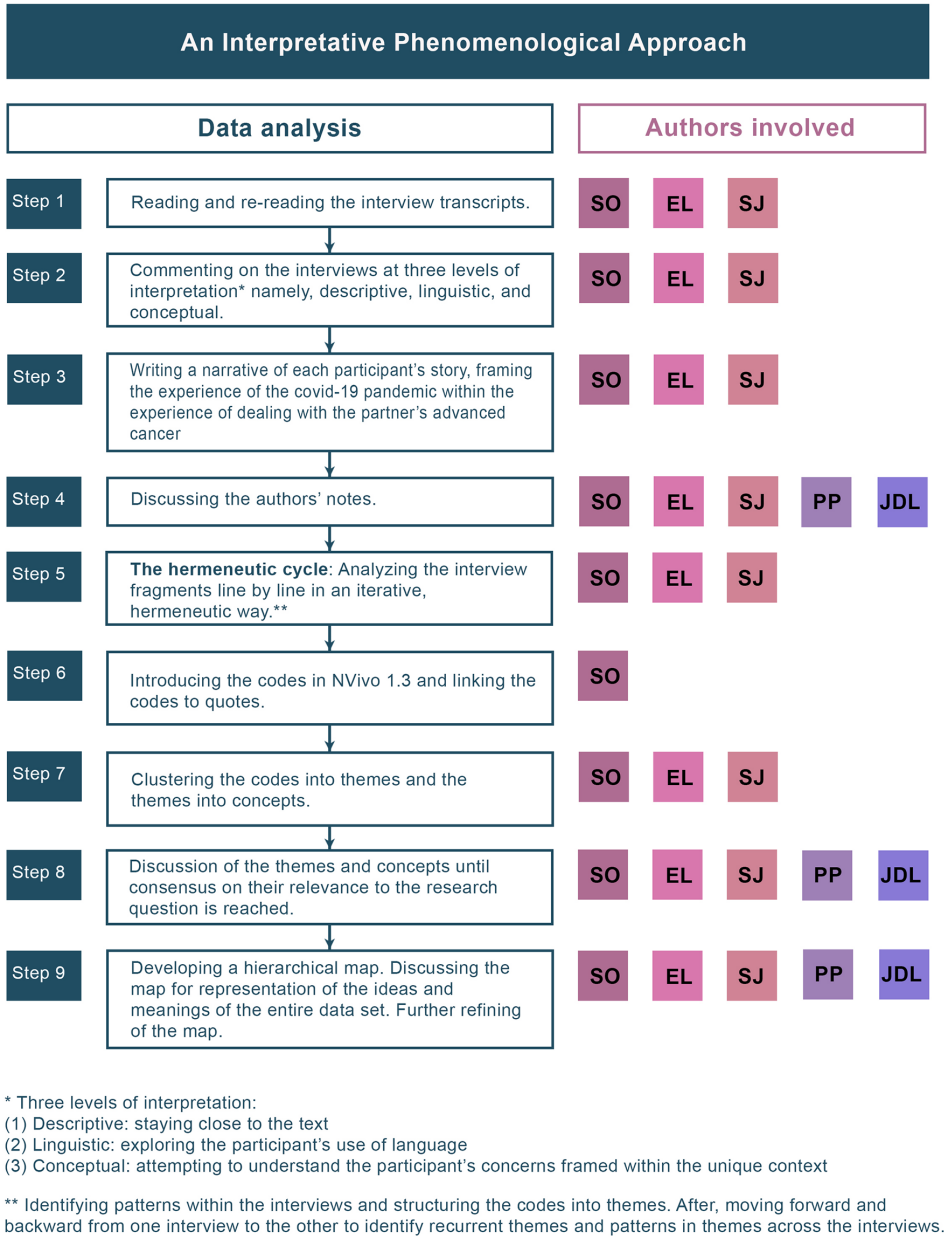
Data analysis and authors’ contribution. *Three levels of interpretation: (1) Descriptive: staying close to the text; (2) Linguistic: exploring the participant’s use of language; (3) Conceptual: attempting to understand the participant’s concerns framed within the unique context. **Identifying patterns within the interviews and structuring the codes into themes. After, moving forward and backward from one interview to the other to identify recurrent themes and patterns in themes across the interviews.

Twelve interviews were analyzed inductively according to the procedure proposed by an interpretative phenomenological analysis.^20^ The interviews were read through several times and were commented on at three levels of interpretation namely, descriptive (staying close to the text), linguistic (exploring the use of language), and conceptual (understanding the participant’s concerns). Each participant’s story was summarized in a narrative style and discussed amongst the authors’ team. The interviews were analyzed in a hermeneutic way, typical for an Interpretative Phenomenological Approach.^20^ A hermeneutic analysis is a cyclical process, by which the researcher moves back and forth through the data, hereby interpreting each part of the interview within the context of the participant’s story and considering the context on its turn being influenced by the different parts.^20^ The interviews were then coded and patterns were identified within the interviews (in-case analysis), and also through an iterative approach by moving through and across interviews (across-case analysis). Consequently, the codes were introduced in NVIVO 1.3 and linked to quotes. Next, the codes were clustered into themes and the themes into concepts. All themes and concepts were discussed within the research team on their relevance to the research question until consensus was reached. Eventually, a hierarchical map was developed, discussed, and refined for representation of the ideas and meanings based on the entire dataset. The concrete steps and author involvement are represented in Figure 1.

### Validity and reliability

Following each interview, a participant debriefing and concise debriefing of the interviewer by her supervisors increased the credibility and reliability. Field notes were made during and immediately after each interview to ensure reflexivity.^22^ In order to ensure trustworthiness and credibility, the analysis was conducted in a structured and traceable way, and the appropriateness of the themes was verified by in-case and across-case analyses, conducted by a collaborative multidisciplinary team.^23^

### Ethics

Approval: Ethical approval was provided by the Ethics Committee Research UZ / KU Leuven on October 4, 2019, study number S63166 and by the Ethics Committee of Ghent University Hospital on October 17, 2019, study number BC-06066.

Reflections on the research team: Besides the first author, the multidisciplinary authors team was compri-sed of one professor in health psychology (EL), who supervised the study; one doctor in medical sciences (SJ); and two professors in primary care (PP & JDL). One author was the principal caregiver of a person who died of cancer and COVID-19. All authors are methodologically experienced in either palliative care research or chronic disease management. The first author, who conducted the interviews, is part of a team of communication trainers at KULeuven and is experienced in communication with people under burdened circumstances considered as psychosocial.

Reflections on the data collection: Interviews were conducted according to the COVID-19 measures in force at that time. The authors were aware of the risks associated with online interviews regarding potentially psychological topics (e.g. technical problems or an unexpected computer sign off). Fortunately, no incidents were reported. All participants provided a written, informed consent and participated voluntarily in the study.

## Findings

Below, we present the findings on how partners experienced COVID-19 while taking care of a person recently diagnosed with advanced cancer. Participant characteristics are presented in Table 1.

**Table 1.** Participants demographic information.

Characteristics of the partners/participants	Total number		12
	Age	18–30	0
		31–40	1
		41–40	1
		51–60	3
		61–65	7
	Gender	Male	4
		Female	8
	Civil status	Married	9
		Living together	3
		Living apart	0
	Education	Secondary school	6
		Graduate degree	
		Bachelor’s degree	2
		Master’s degree	3
		Doctoral degree	1
	Number of children living at home	0	9
		1	2
		2	1
		>2	0
	Moment of interview*	March–May 2020^(1)^	2
		June–October 2020^(2)^	4
		November 2020–March 2021^(3)^	8
	
Characteristics of the patients	Age of cancer diagnosis	Median (range)	58.5 (35–77)
	Age of advanced cancer diagnosis	Median (range)	59.5 (35–80)
	Time between diagnosis of advanced cancer and start of the pandemic	Median (range)	2.5 months (0–8 months)
	Gender	Male	8
		Female	4
	Education	Secondary school	4
		Graduate degree	2
		Bachelor’s degree	2
		Master’s degree	3
		Doctoral degree	1
	Major cancer diagnosis	Breast cancer	2
		Colon cancer	1
		Glioblastoma	1
		Head and neck cancer	1
		Hodgkin lymphoma	1
		Kahler myeloma	1
		Lung cancer	1
		Lung neuro-endocrinal tumor	1
		Lung pleura cancer	1
		Merkel cell carcinoma	1
		Non-Hodgkin Lymphoma	1

* COVID-19 measures at the moment of interview:

(1) First lockdown: Only essential services are allowed; shops, hairdressers, schools etc. are closed. Gathering of people is forbidden. Traveling is impossible, national borders are closed. Reorganization in the hospitals: only emergencies and COVID-19 patients are allowed. Family doctors work via telemedicine where possible. There is a shortage of personal protection materials.

(2) Gradual relaxation of the measures: Schools reopen half of the week, shops are open with limited capacity, non-essential services are allowed, consultations with the doctor is possible for urgent and planned care, telework remains the standard. Gatherings outside of up to four people are allowed. Outdoor sports are allowed. Restaurants and bars reopen with limited capacity. Terrasses are open. Youth movements can have their summer camps outside in bubbles of 50 persons.

(3) Second lockdown: non-essential shops are closed, restaurants and bars are closed, visits are limited to one person at a time, and no more than one person per family member (always the same person). Outdoor activities of up to four persons are allowed. Schools are open half-time. All courses at universities and university colleges are online. A curfew is set from midnight to 5 a.m.

### Being challenged by two different, potentially traumatic events at a time

The idea of coping with two simultaneously potentially traumatic events was visible in the data. First, as the caregiver’s partner was recently diagnosed with advanced cancer, participants mentioned being confronted with the threat of their partner’s impending death. Second, participants feared that COVID-19 could cause an untimely death of their partner. Moreover, all persons involved, were considered to be a life-threatening danger by exposing the patient to the virus. Surprisingly, none of our participants reported any fear of becoming severely ill themselves.

*Because of the Coronavirus, everything is way more complex. Naturally, he can’t see people. But I have to do the shopping anyway. I can bring it [the coronavirus] in here. The kids can bring it in too. If he gets infected, his life is in shreds. (P7-female-54y)*

### Living in a double cage

Overall, we can say that almost all participants experience the COVID-19 pandemic as living in a double cage. Where it previously proved difficult to find ways to escape the limitations in daily life imposed by advanced cancer, COVID-19 now appears to limit these possibilities even further.

*But well, yes, uh, you’re in a cage, so to speak. You’re in the cage of the disease and you’re in an extra cage that’s around it and that’s that COVID one. (P11-male-63y)*

#### No escape

As a result of COVID-19, both the carer and the patient were forced to adapt to the new reality of everyday and leisurely activities. All public venues offering entertainment were closed and restrictions made it impossible to travel. These mandates affected patients from accomplishing those items on their bucket list, the events and adventures one hopes to experience during their lifetime.

*Nobody comes over anymore. That makes it all really difficult, you know. There is no, how should I say this, no distraction anymore. (. . .) We used to go to museums and to the theatre. We made trips. We love being in the Ardennes, where I always feel better and have the time to compose myself. But it’s all impossible now. A lot has been taken away from us, we get nothing in return, except for too much time to think [about the cancer]. (P6-female-62y)*

Those partners accustomed to going to work everyday suddenly found themselves working remotely from home during the pandemic. Adapting to this new environment often imposed an even further burden.

*I work from home now and my husband is there too, so, yeah . . . That’s an advantage on the one side and a disadvantage on the other, you know. I can be there for him 24 hours a day, seven days per week, but on the other side, I’m never away from him [and the cancer] anymore, you see. (P7-female-54y)*

#### Time passes at different speeds

During the pandemic, the partner’s lifestyle slowed dramatically while the pace of the patient’s cancer continued without impediment, leading to an awareness that the patient’s life may come to a close before the COVID-19 measures are lifted.

*If there would be a vaccine next year, a good vaccine I hope, then you can say, last year was just a year, it doesn’t matter. But for her [the patient], that year was a year meant to enjoy things and then it ends like this. While for me, that year, yeah, is just a moment, something that happened by coincidence. It is what it is. And it is the same for you and for me, for everybody. But for her, it was a year that we could have been doing pleasant things. (P8-male-62y)*

### Benefits

Despite the difficult circumstances of COVID-19, some partners report finding benefits due to the pandemic itself. As such, the shared, mutual experience often creates a high level of connectedness with others. Some participants even expressed their feelings of enhanced appreciation for what prior had been taken for granted. The lockdown measures resulted in more time at home, which afforded more quality time with the family and provided an opportunity to improve the relationship between patient and partner. In addition, as daily schedules became more flexible, stress levels tended to be less acute. In such an environment, people became more creative in everyday tasks.

*I try to cook extra tasty meals because he has to eat well. (. . .) And yes, now that I also have more time [because of the COVID-19 measures], I spend more hours in the kitchen preparing something extra. So, . . . we enjoy those small things. For instance, being together in the garden whenever the weather is good. We’ve had that luck now. Then we work together in the garden. We had never done that before. (P2-female-57y)*

### Challenges to resilience

When confronted with a partner’s diagnosis of advanced cancer, many caregivers seem to succeed in recovering from this traumatic stress by building resilience.^24^ This process is promoted by the carer’s individual characteristics, called ego-resiliency, and the availability of contextual factors promoting resilience.^10,14,24^ However, both ego-resiliency and the resilience promoting context support can be strained by the COVID-19 pandemic and its measures.

#### A challenged ego-resiliency

The COVID-19 pandemic and its accompanying mandates challenge the ego-resilience of the participants. Here, three components of ego-resiliency—balanced dependency, positive attitude, and the ability to maintain control over the incoming and outcoming cancer-related information—all come under pressure.

*Balanced dependency* involves a mutual give and take between carers and those on whom they can rely. This characteristic ensures that partners are willing to ask for and accept help whenever needed.^9,10,14^

However, COVID-19 measures hinder the accessibility to professional help as partners can no longer join the patient for their medical visits. Likewise, informal practical help is no longer available during the lockdown. Since both professional and informal help are systematically weighed against the risk of infection, an information void can develop.

*And then again, that insecurity. That’s, yeah, you can’t visit the GP. He [the GP] doesn’t want to see anyone because of the virus. And you don’t want to take the risks either. (P5-male-47y)*

On the other hand, although technology is a useful tool, it can never replace in-person visits.

*What we do now, is video talking. But that’s not like the family discussions we used to have. That’s just seeing each other and having some small talk. Or we eat a cake together, on Saturdays, at four PM when the kids have their fruit porridge and then we eat our carrot cake. Such stupid things, but that’s not that family council talk anymore, no. (P4-female-62y)*

*A positive attitude* to life refers to the mental state of being optimistic about the events in one’s life and establishing a mindset that allows one to look forward to a prosperous future. It helps people solve their problems and attach positive meaning to a crisis.^10,14^ However, in the midst of a pandemic, it is difficult to stay optimistic since even positive events can take on a negative connotation.

*Of course, we had to come back as soon as possible because Jordan was going to close its airspace. My husband definitely had to come back home. We moved heaven and earth [to book a flight back home]. I was scared to death. I was so afraid that we wouldn’t make it in time. It would have meant that my husband wouldn’t have survived it, I’m afraid. (P6-female-62y)*

*Having control over the information* the partners receive and want to communicate to others is resilience-supporting when dealing with advanced cancer.^14^ However, participants discussed their frustration at not being wholly informed due to the prohibition of accompanying the patient to hospital visits and treatments. This often led to a feeling of inadequacy and uncertainty. Electronic communication is more direct and may urge the partner to undertake difficult discussions with family and friends about the cancer evolution, prognosis, and therapy. Nevertheless, some partners reported that phone calls provided an opportunity to escape from such difficult conversations.

*I would have told them [friends] everything in person [about the advanced cancer]. I think that’s important. But now it’s all different, you see. But you can’t keep everything a secret [the cancer is evolving badly]. So, I tell them something [it’s not going well], not into details, only the essential part. (P10-female-61y)*

#### A supporting context at risk

Recognizing *the patient as vulnerable* allows for the carer to better deal with the risks posed by the novel virus. People with cancer are considered at high risk for severe illness when infected with the SARS-COV-2 virus. Healthcare professionals, family, and friends who scrupulously follow all the COVID-19 measures are found to be the most supportive.

*If people show. . .[that they respect all the rules to prevent infection], they express: “I love you” and they don’t want you to get sick. They want . . .[the patient to stay alive]. So, I think this is fantastic. Yes, I think this is great. But, on the other side, it confronts you again and again with the facts, but, no, really, it’s great to see that people respect you and take responsibility. Apparently, they don’t want to lose him either. (P12-female-63y)*

*The recognition of the partner in a caregiving role* is also significant to resilience promotion. Partners who desire to be involved in the cancer process from diagnosis to caregiving and treatment discover that their role is supportive and respected. This role, however, has been threatened by the COVID-19 emergency due to the prohibition against hospital visits.

*Meaningful relationships* usually are essential in leading the carer through the coping process in the case of advanced cancer.^14^ Caregiver-patient relations under the pandemic often take on a new meaning. For example, most partners mention isolation, loss of connectedness, and an absence of physical affection. of connectedness with others and the difficulties they experience with the loss of physical affection.

*On Monday evening, I needed a shoulder to cry on. I have a friend who comes over every day, but he is no longer allowed to come in. So, I took my car and drove to his house. And I went there to cry. We stayed in the garden, of course. And yes, it was worth it. (P11-male-63y)*

*The permanent availability* of social support networks is paramount in building resilience.^14^ Unfortunately, this availability is seriously threatened by the lockdown measures and out of fear of infecting the patient. For instance, a partner discusses how his friends used to bring food to him, but this stopped out of the fear of infection.

*Before the pandemic, it was natural that friends and family came over to mow the grass or clean the windows. Every now and then they put fresh meals at the front door. That all seemed so natural. While now, it’s Covid and everyone stays away. Moreover, everybody is afraid to bring a dish over here with who knows what of their lives in it while yes, there is now a very sensitive person walking around here. (P7-female-54y)*

### The COVID-19 pandemic as a catalyst for coping strategies

Although the COVID-19 emergency has placed pressure on some resilience-promoting elements, the interview data reflect adaptive coping.

#### Focusing on daily life

While some partners mention the ease of adapting to a new normality, others reflect the active search for alternative means to maintain daily routines altered by the measures and mandates set down by the pandemic. The following illustrates how everything is balanced against the risk of infection.

*And yeah, we stay home now. Before, you went out working, you were away [from home and the cancer] from the morning until the evening. And then on Saturdays and Sundays, we went for a bike ride or . . . But now with the Corona virus, we go walking too. The usual life. (P9-female-62y)*

#### Taking responsibility

The limited availability of healthcare professionals, family, and friends and the permanent awareness of the infection risk, stimulates the partners to assume new roles. Meanwhile, a shift in responsibility comes to the fore as the care for the patient is enhanced while responsibility for oneself and others becomes less crucial.

*We’ve had it up to here with Corona. Everybody who could leave the hospital, was discharged, go, go, go . . . And he couldn’t walk yet, but he could come home anyway. So, for me it felt like: yes, hooray, we are going to be together again. And we asked the physiotherapist to come over. But she didn’t have time, or she was not allowed to do home visits or . . . and then I had to take over as his physiotherapist. I was really scared, what if he falls? But everything turned out well. (P4-female-62y)*

#### Managing the situation

The pandemic and its constant threat to the lives of the patients stimulate the partners in managing the cancer by controlling the risk of infection, and hence, in their perception, enhancing the chances of surviving. However, managing a situation with two potentially traumatic events requires much more self-confidence.

*The only thing, during the first lockdown, was that my husband could no longer get his lymph drainage because the hospital was closed. Hence, his leg started to swell again, and he had more pain. (. . .) And I fell a bit helpless and even a bit angry. I said, excuse me, but there are people here with other problems than Covid. I agree, it [the pandemic] ’s gigantic, it’s a disaster, but there are other problems too. And then, I have . . . pushed, may not be the best word, but at least, I’ve said: you should contact the physiotherapist. In the end, they have allowed people with that kind of pathology to the hospital, and he could have his lymph drainage. (P10-female-61y)*

#### Mastering the situation

Partners who master the situation in which they find themselves, accept it and flexibly adjust their lifestyles. The COVID-19 emergency requires partners to face some harsh realities such as the impending death of the patient. Despite the COVID-19 measures, the participants have succeeded in mastering the situation, albeit in a way that is balanced against the risk of infection. Therefore, cherished moments are created and social contacts are renewed more imaginatively.

*In summer, we allowed some friends to visit us. We re-arranged the garden table and we were sitting as the king and queen each at one end of the table. That was with 1,5 m in between the two of us. We were sitting outside, in the sun. In that way, we could eat together and talk all day. (P7-female-54y)*

## Discussion

### Main findings

Having a partner recently diagnosed with advanced cancer is a psychological hardship, and building resilience is the only way to escape.^14^ When confronted with the COVID-19 pandemic, carer resilience is further challenged, and as such is seen as being “trapped in a double cage.” In light of the crisis, resilience assumes a more nuanced definition.

In line with the findings of Radcliffe et al.^25^ and Chia et al.,^26^ our data reveal that the COVID-19 pandemic magnified the vulnerability of the resources (ego-resiliency and the availability of a social support network) available to ensure a resilient process.^25,26^ Nevertheless, as also confirmed by Radcliffe et al. and by Chia et al., different coping mechanisms such as maintaining normality,^25,26^ assuming responsibility^26^ and managing^25^ and mastering the situation are stimulated. Furthermore, as affirmed by Radcliffe et al., some carers discuss general positive aspects of the pandemic resulting in a resilient process being promoted overall and a new equilibrium being established.^25^ However, we could not confirm some of the findings of Sia et al. that are probably related to cultural habits, as there are, COVID-19 being less imminent as cancer or the downplaying of risks.^26^ Nor could we confirm the finding of Radcliffe et al.^25^ and Sia et al.,^26^ stating that the carers exposed a sense of trust in authorities and healthcare providers. Our findings emphasize the dynamic features of the resilience process as conceptualized by Bonanno et al.^13^ and as applied to cancer caregivers by Opsomer et al.^10,14^ Indeed, although important elements of resilience are strained, others are stimulated or flexibly adjusted to the new situation. It is also visible that our data did not reveal evidence on the occurrence of inner strength and flexibility among participants in dealing with diagnosis and the pandemic. However, we believe it to be very likely that these characteristics present themselves through coping strategies as expressed by the participants. What has come to light, though, is the ease with which partners seemed to adapt to the crisis. For instance, they accepted the pandemic and its imposed measures, continuously balanced the risk of infecting the patient against their needs to maintain wellbeing, and taking control over the communicative process regarding the cancer. Consequently, carers succeeded in adaptive coping with the cancer diagnosis during this pandemic emergency by using the same coping strategies as in coping with advanced cancer only,^14^ albeit in a balanced and more inventive way.

In sum, our findings point out that important resilience-supporting characteristics—balanced dependency, being the information processor, and positivity—are at risk when dealing with two potentially traumatic events at the same time. In addition to individual characteristics, some contextual features (e.g. the availability of professional and personal support) can no longer be guaranteed. Consequently, mental distress can increase, resulting in coping strategies closely related to fight and flight reactions people experience in situations perceived as threatening.^27^ According to the general adaptation syndrome (GAS) model of stress, three stages can be distinguished, namely the alarm phase, the resistance phase, and the exhaustion phase.^28^ The intense fear of infecting the patient coupled with the troubling idea of being trapped in a double cage, reflect the alarm phase. Consequently, one will attempt to cope with the situation and to manage the stressors. This behavior can explain our participants’ inventive ways of coping with escape from the threat.^28^ However, one should be aware of the phase of exhaustion that may follow. It can be supposed that resilience-promoting contextual features such as availability of the supporting context and meaningful relationships should be preserved to increase opportunities of a resilient outcome based on sustainable coping strategies.^17,19,29^

### Strengths and limitations

The unforeseen outbreak of COVID-19 at the start of our longitudinal study on resilience in partners of persons diagnosed with advanced cancer offered us the opportunity to extend our study by exploring resilience in the unique situation of being challenged by two independent, potentially traumatic events at a time. The COVID-19 emergency challenged the participants’ resilience and accentuates the strengths and the flaws of the resilience process through the lens of a magnifying glass. The use of an interpretative phenomenological approach, characterized by an iterative analysis cycle, allowed us to investigate our participants’ lived experiences thoroughly.^20^ Moreover, the interdisciplinary composition of our research team (including an author with first-hand experience) and the emphasis on teamwork can enhance the trustworthiness and validity of our findings.^22^

This study, however, was also subject to some limitations. Our sample was purposely selected from interviews of participants in a study on resilience. It cannot be excluded that partners who are the most successful in building a resilience process are more willing to participate in a study oriented to positive psychological developments. Consequently, a sample bias could have influenced the rather positive results regarding the coping strategies. Moreover, all our participants were from western Europe. Cultural influences could explain the contradictory findings between our study and other study results.^26^ Besides, we cannot expand our findings to other situations where successive potentially traumatic events challenge the caregiver. The resilience framework developed by Bonanno et al.^13^ and applied to advanced cancer caregiving by Opsomer et al.,^10,14^ holds up when a community threat crosses the resilience process in progress, and challenges the caregiver’s resilience as a second, potentially traumatic event. However, more research is needed to explore the effects of two or more successive potentially traumatic events and of any cumulative combination on outcomes over time.

### Implications

To the best of our knowledge, our study is the first to explore what it means to be challenged with a second potentially traumatic event coupled with a recent diagnosis of advanced cancer or cancer in a palliative stage. The COVID-19 crisis included a second major threat over and above the challenge of dealing with advanced cancer. Its distinctive nature compared to stressors that more readily occur in daily life, reveals new insights on the resilience process. To guide partners of people with advanced cancer in developing a resilience process throughout cancer caregiving, healthcare professionals should be aware of the impact of any second potentially traumatic event on existing resilience promoting characteristics and context features. Furthermore, healthcare professionals should look for a way to bring to the forefront those characteristics and features that are still intact. In dealing with major stressors, partners seem to employ an array of inventive and balanced coping strategies. These strategies, predominantly personal in nature, seem to set the stage for a resilient outcome, even in the presence of extreme environmental constraints (e.g. sheltering measures during this pandemic). Nevertheless, permanent support by health services and professionals should be guaranteed, albeit technology-driven whenever face-to-face contact is not possible by the health measures imposed. Policy makers should also be aware of the importance of availability of social support networks in building and maintaining resilience in carers of patients with advanced cancer. Although it requires creativity and inventiveness, it is necessary to guarantee the opportunity to meet with people.

Future studies should further reveal how this dependency on personal strengths evolves over time and whether, when, and how there might be a risk for exhaustion.

### Conclusion

Partners of those diagnosed with advanced cancer perceive the pandemic as “living in a double cage” with no way to escape the cancer nor the COVID-19 threat. Due to pandemic mandates and restrictions, the pace of their lives slows. However, COVID-19 does not slow the progression of the cancer, nor does it allow for an escape from the cancer. Some resilience promoting characteristics may be strained due to the intense fear of the partner infecting the patient and to the emergency restrictions put in place, while other characteristics are redefined, and flexibility and inner strength even seem to be reinforced. The latter two seem to be linked to creative and balanced ways of coping with both the advanced cancer and the COVID-19 pandemic. Some partners even report finding benefits during the COVID-19 crisis, meaning that a resilience process is initiated. However, risk for exhaustion is realistic possibility.

## Supplemental Material

sj-pdf-1-pmj-10.1177_02692163221080660 – Supplemental material for Trapped in a double cage: How patients’ partners experience the diagnosis of advanced cancer in times of the COVID-19 pandemic: An interpretative phenomenological analysis Click here for additional data file. Supplemental material, sj-pdf-1-pmj-10.1177_02692163221080660 for Trapped in a double cage: How patients’ partners experience the diagnosis of advanced cancer in times of the COVID-19 pandemic: An interpretative phenomenological analysis by Sophie Opsomer, Sofie Joossens, Jan De Lepeleire, Peter Pype and Emelien Lauwerier in Palliative Medicine

sj-pdf-2-pmj-10.1177_02692163221080660 – Supplemental material for Trapped in a double cage: How patients’ partners experience the diagnosis of advanced cancer in times of the COVID-19 pandemic: An interpretative phenomenological analysis Click here for additional data file. Supplemental material, sj-pdf-2-pmj-10.1177_02692163221080660 for Trapped in a double cage: How patients’ partners experience the diagnosis of advanced cancer in times of the COVID-19 pandemic: An interpretative phenomenological analysis by Sophie Opsomer, Sofie Joossens, Jan De Lepeleire, Peter Pype and Emelien Lauwerier in Palliative Medicine
