# Supplementary material for: Trapped in a double cage: How patients’ partners experience the diagnosis of advanced cancer in times of the COVID-19 pandemic: An interpretative phenomenological analysis
Source: Palliat Med. 2022 Mar 10;36(5):810–20. doi: 10.1177/02692163221080660 (PMC9087315; doi:10.1177/02692163221080660)
Supplement: sj-pdf-1-pmj-10.1177_02692163221080660 – Supplemental material for Trapped in a double cage: How patients’ partners experience the diagnosis of advanced cancer in times of the COVID-19 pandemic: An interpretative phenomenological analysis [file sj-pdf-1-pmj-10.1177_02692163221080660.pdf]

Supplement 1. Code tree and data saturation table

Concept	Theme	Code	Int. 1	Int. 2	Int 3	Int. 4	Int. 5	Int. 6	Int. 7	Int. 8	Int. 9	Int. 10	Int. 11	Int. 12
**Double trouble**	The first potentially traumatic event	The participant's partner diagnosed with advanced cancer	**X**	x	x	x	x	x	x	x	x	x	x	x
	The second potentially traumatic event	Not only the SARS-CoV-2 virus but everybody including the partner can be life-threatening for the patient	**X**	x		x		x	x	x	x		x	
**Impact on ego-resiliency**	Balanced dependency	Both professional and informal help are weighed against the risks of infection					**X**		x	x				
		Dependency on technology	**X**			x		x	x		x		x	x
	Positivity	Positive events take on a negative connotation						**X**						
	Being the information processor	Information is no longer available	**X**	x		x						x		
		An escape from difficult talks				**X**					x			
		Less secrecy									**X**			x
**Impact on the resilience-supporting context**	Being seen and known	Recognized vulnerability		**X**			x						x	x
		Being recognized in the partner role	**X**	x		x	x	x				x		x
	Meaningful relationships	Isolation		**X**		x	x	x			x	x	x	x
		Social contacts are highly appreciated	**X**	x	x									
	Availability	Covid measures hinder availability	**X**			x			x					
**Living in a double cage**	time passes at different speeds	COVID-19's pace versus cancer's pace	**X**	x	x	x		x		x		x		
		Work goes on but leisure time stops		**X**										

	No way to escape	Escaping from the cancer is no longer possible		X	x	x		x	x	x	x	x	x	x
**Finding benefits**	Benefits ascribed to COVID-19	More quality time		X	x		x		x					
		Better partner relationship	X				x							
		Enhanced creativity		X		x				x				
**A catalyst for resilience coping strategies**	Taking up responsibility	Taking up new roles	X			x	x	x	x	x				
		Balanced responsibility		X			x		x					
	Focusing on daily life	Life with cancer becomes normal			X	x				x	x	x		
		Stimulated creativity		X		x				x				
		Balanced normality								X				
	Managing	Controlling life by controlling the risk of infection					X			x				
		Control is stimulated						X				x		
		Managing using creativity				X	x					x		
	Mastering	Accelerated acceptation				X		x						
		Balanced mastering	X	x	x	x	x	x					x	
		Mastering through creativity			X	x			x		x			
		**New codes**	**11**	**8**	**2**	**3**	**2**	**2**	**0**	**1**	**1**	**0**	**0**	**0**
