# Supplementary material for: Trapped in a double cage: How patients’ partners experience the diagnosis of advanced cancer in times of the COVID-19 pandemic: An interpretative phenomenological analysis
Source: Palliat Med. 2022 Mar 10;36(5):810–20. doi: 10.1177/02692163221080660 (PMC9087315; doi:10.1177/02692163221080660)
Supplement: sj-pdf-2-pmj-10.1177_02692163221080660 – Supplemental material for Trapped in a double cage: How patients’ partners experience the diagnosis of advanced cancer in times of the COVID-19 pandemic: An interpretative phenomenological analysis [file sj-pdf-2-pmj-10.1177_02692163221080660.pdf]

Supplement 2: Interview guide

1. Can you tell me what happened to you after you got the diagnosis of advanced cancer? How did you feel? How did you react?
2. What helps you cope with the diagnosis?
3. How did you inform your family and friends (about the partner's cancer diagnosis)?
4. How did people react to the diagnosis? What did this mean to you?
5. How were you informed about that type of cancer?
6. Did you ask someone (family, friends, healthcare professionals) for help? Can you tell me more about this?
7. How do you envision the future? What expectations do you have?
8. Did the cancer diagnosis alter your (partner) relationship?
9. Where do you get satisfaction from?
10. What's the most difficult part in caregiving?
11. Do you see any positive aspects in caregiving?

Covid-19 related supplementary questions (only posed when the partner mentioned the Covid-19 pandemic)

a. What did it mean to you not to have personal contact? How did you handle this?
b. What did it mean to you not being allowed to join your partner in the hospital?
c. What did it mean to you that nobody could come to your home to help you?
d. Do you see positive aspects in the Covid-19 pandemic?

Probes

1. Can you tell me more about this?
2. Can you give an example of this?
3. What did this mean to you?
4. How did you react then?
5. How did you solve that?
